# Identifying scenarios and risk factors for Q fever outbreaks using qualitative analysis of expert opinion

**DOI:** 10.1111/zph.12923

**Published:** 2022-03-03

**Authors:** Tabita Su‐En Tan, Marta Hernandez‐Jover, Lynne Maree Hayes, Anke Katrin Wiethoelter, Simon Matthew Firestone, Mark Anthony Stevenson, Jane Heller

**Affiliations:** ^1^ Gulbali Institute Charles Sturt University Wagga Wagga Australia; ^2^ School of Animal and Veterinary Sciences Charles Sturt University Wagga Wagga New South Wales Australia; ^3^ Faculty of Veterinary and Agricultural Sciences The University of Melbourne Parkville Victoria Australia

**Keywords:** *Coxiella*, disease outbreaks, disease prevention, Q fever, qualitative research, risk factors

## Abstract

Q fever is an important zoonotic disease perceived to be an occupational hazard for those working with livestock. Outbreaks involving large numbers of people are uncommon, but the increasing case incidence coupled with changing environmental and industry conditions that promote transmission of Q fever has raised concerns that large and serious outbreaks could become more frequent. The aim of this study was to use expert opinion to better understand how large Q fever outbreaks might occur in an Australian context and to document factors believed to be drivers of disease transmission. Focus groups were conducted with human and animal health professionals across several Australian states. All discussions were recorded, transcribed verbatim and imported into NVIVO for thematic analysis. Four anthropogenic risk factors (disease awareness, industry practices, land use, human behaviour) and three ecological risk factors (physical environment, agent dissemination, animal hosts) emerged from the data. Analysis of expert opinions pointed to the existence of numerous scenarios in which Q fever outbreaks could occur, many of which depict acquisition in the wider community outside of traditional at‐risk occupations. This perception of the expansion of Q fever from occupational‐acquisition to community‐acquisition is driven by greater overarching economic, political and socio‐cultural influences that govern the way in which people live and work. Findings from this study highlight that outbreaks are complex phenomena that involve the convergence of diverse elements, not just that of the pathogen and host, but also the physical, political and socioeconomic environments in which they interact. A review of the approaches to prevent and manage Q fever outbreaks will require a multisectorial approach and strengthening of community education, communication and engagement so that all stakeholders become an integrated part of outbreak mitigation and response.

**Impacts**

- The risk of large Q fever outbreaks could increase because of changing environmental and industry conditions that promote transmission.
- Analysis of expert opinions suggest numerous scenarios in which Q fever outbreaks could occur, many of which depict acquisition in the wider community outside of traditional at‐risk occupations.
- Approaches to prevent and manage Q fever outbreaks will require a multisectorial approach and strengthening of community engagement in outbreak mitigation and response.

## 1 INTRODUCTION

Q fever is a zoonotic disease caused by the rickettsia‐like organism *Coxiella burnetii*. It has an extensive range of natural host reservoirs, including wildlife, domestic and feral mammals. Domestic ruminants (cattle, sheep and goats) are considered the primary sources of human infection and Q fever is commonly perceived as a hazard for persons working in livestock industries (Maurin & Raoult, 1999; Roest et al., 2011). *Coxiella burnetii* is highly infectious for humans with the inhalation of aerosols or dust contaminated with animal secretions, including faeces, urine and birth products, being the main transmission route (Brooke et al., 2013; Eastwood et al., 2018; Sawyer et al., 1987). Drought conditions raise the risk of Q fever infection because dry dusty environments and increased livestock movements aid dispersal of the bacteria to populations who have not been previously exposed (Gidding et al., 2009). The ability of *C*. *burnetii* to persist in the environment for prolonged periods means that people can be infected from contaminated environments even in the absence of animal contact (Australian Technical Advisory Group on Immunisation (ATAGI), 2018; Kersh et al., 2013; Raoult & Marrie, 1995).

Q fever is a health concern in many countries because infection in humans can lead to chronic and debilitating disease (Angelakis & Raoult, 2010; Hechemy, 2012). Reported outbreaks of Q fever are generally sporadic and transient, but the large and sustained epidemic in The Netherlands from 2007 to 2010 has demonstrated that Q fever has the potential to become a major public health threat (Delsing et al., 2010; Roest et al., 2011). More than 4,000 human cases from multiple municipalities were notified across the south of The Netherlands and traced back to dairy sheep and goat farms as the source of infection (Schneeberger et al., 2014). The large number of infections from this outbreak occurring in the community outside of traditional at‐risk workplaces has expanded the view of Q fever as an industry related disease to a wider public health issue.

In Australia, Q fever is the most common of the direct notifiable zoonoses with an annual notification rate of 2.1 cases per 100,000 population in 2017 (Australian Government Department of Health, 2017). The reported incidence of Q fever is relatively high in Australia (1.5 to 4.9 cases per 100,000) compared to European countries (0.18 cases per 100,000) and the USA (0.04 cases per 100,000) (Communicable Diseases Network Australia, 2018; Rahaman et al., 2020). The distribution of Q fever in Australia is geographically uneven with ‘hotspots’ in central Queensland and around the New South Wales‐Queensland border (>13 cases per 100,000) (Australian Technical Advisory Group on Immunisation (ATAGI), 2018). In addition to livestock, exposures to native wildlife, particularly kangaroos and wallabies, have been associated with human infection, underscoring their role in the epidemiology of Q fever in Australia (Clark et al., 2020; Clutterbuck et al., 2018; Graves & Islam, 2016). A small but notable number of reported human cases did not have animal contact or lacked known risk factors for acquisition (Clutterbuck et al., 2018; Graves & Islam, 2016; Rahaman et al., 2020; Sloan‐Gardner et al., 2017). A nationally funded Q fever vaccination programme (2001–2006) that targeted high‐risk occupations connected to the livestock industry was successful at reducing case numbers, especially in abattoir settings (Gidding et al., 2009). A growing number of groups outside of the livestock industry are recognized to be at risk of Q fever and vaccination is also recommended for people living in proximity to farms, stockyards, meatworks, along livestock transport routes and visitors to livestock facilities (Communicable Diseases Network Australia, 2018). However, since 2009 the number of notifications of Q fever in Australia has risen for reasons that are unknown (Clutterbuck et al., 2018; Lowbridge et al., 2012).

Q fever outbreaks in Australia are usually related to occupational or environmental exposure and limited to several cases, very occasionally affecting several dozen people (Eastwood et al., 2018). However, Australia's rebounding high case incidence coupled with the rising intensity of drought conditions and non‐occupational exposures that promote the transmission of Q fever beyond rural populations has raised concerns that large and serious outbreak events could occur here with increased frequency. The epidemiology of Q fever is incompletely understood and outbreaks around the world continue to occur in surprising ways. Appraising the complex transmission pathways and shifting epidemiological conditions in Australia can serve as a valuable example of the changing Q fever risk profiles that could occur elsewhere. Previous studies of the epidemiology of Q fever in Australia have focused on ecological mechanisms, namely disease distribution and reviews of human cases for direct risk factors for infection. Detailed investigatory studies assessing how hosts and the pathogen intersect with factors broadly classed as ‘environmental’ (including the geographical distribution of at‐risk populations, which in turn, is influenced by economic and social factors) are lacking (‘Epidemiology is a science of high importance’ [Editorial], 2018). With this background, the aims of this study were to: (a) use expert opinion to better understand how Q fever outbreaks are perceived to occur in Australia; and (b) provide commentary on how various factors might drive Q fever transmission and contribute to future outbreaks.

## 2 MATERIALS AND METHODS

### 2.1 Research design

A focus group exercise was designed to explore underlying assumptions, considerations and knowledge surrounding large Q fever outbreaks in Australia. In brief, experts participated in a moderated discussion to provide a probability estimate that the source of an outbreak affecting more than 25 people (considered a large outbreak in Australia) was from a list of species including cattle, sheep, goats, dogs, cats and wildlife. Geographical or epidemiological descriptors were deliberately omitted so that participants would have the freedom to consider a wide range of factors and possible situations influencing the numerical estimate of species‐specific risk.

Focus groups were conducted in different states to represent geographical differences in participant familiarity with Q fever across Australia. Informed consent was obtained from each participant. This study was approved by the Human Research Ethics Committee, Charles Sturt University, Wagga Wagga, NSW, Australia (Protocol number H19053).

### 2.2 Participant selection and recruitment

Experts were professionals knowledgeable about Q fever and experienced with human, animal or environmental health systems. Inclusion criteria included research or working experience either investigating, diagnosing or controlling Q fever in human and/or animal populations. Using past publications of Q fever in Australia and knowledge of state and federal public health and animal health structure, we aimed to have at least one expert participant from each representative group. Experts were identified through the research team's established professional networks and through snowball sampling where identified experts suggested other suitable participants from amongst their associates (Biernacki & Waldorf, 1981). Participants were selected to ensure that there was a balanced representation at each focus group from human, animal and environmental health.

Experts were approached by email or telephone and invited to attend a focus group session. It is ideal to have 5–8 people per focus group to optimize discussion (Nagle & Williams, 2013) and more experts than the minimum number required were invited to ensure adequate attendance. Several follow‐up emails were sent after the initial invitation to maximize the participation rate.

### 2.3 Data collection

A total of twelve focus group meetings were conducted between March 2019 and January 2020 with a multidisciplinary group of human, animal and environmental health professionals from Melbourne (in Victoria), Perth (in Western Australia), Hunter New England and Sydney (in New South Wales), Brisbane, the Darling Downs and Townsville (in Queensland) and Hobart (in Tasmania). Focus groups were held face‐to‐face in function rooms of major research institutions or universities in Victoria, Western Australia and Queensland. Online focus groups were conducted using teleconferencing facilities for experts in New South Wales, because experts were geographically dispersed, and for Tasmania, because the meeting was organized at short notice.

At each focus group meeting, experts were organized into small groups of 5 to 8 people, comprising equal numbers of individuals with animal health and human health/environmental health expertise, and moderated by a member of the Q fever research team. The task of group moderators was to keep participants engaged and to ensure discussions were on track and productive. A worksheet was distributed to each group describing the hypothetical outbreak scenario. Participants were asked to record minimum, maximum and most likely probability that each species was the outbreak source on a linear scale, the level of confidence (out of 10, with 10 being most confident) probability estimate and to list and rank up to five risk factors that influenced their estimate. A copy of the worksheet template is provided in the supplementary material that that accompanies this paper.

The length of the group discussions ranged between 60 to 105 min. All conversations were recorded with hand‐held digital devices or with video conferencing recording facilities. Audio recordings were transcribed verbatim by a professional transcription service. Returned transcripts were reviewed alongside the audio recordings by a member of the research team (TT) to ensure accuracy. Only minor grammatical adjustments were made for ease of reading. Personal identifiers were removed from the transcribed files to ensure participant responses remained anonymous. Data collection was ceased when TT, who reviewed the recordings from all the focus groups, judged that the range of perspectives appeared to be sufficiently covered and additional data did not generate any substantially new information.

### 2.4 Data analysis

All focus group discussion transcripts were imported into NVIVO 12 for Windows (QRS International, Australia). The method of inductive thematic analysis described by Braun and Clarke (2013) was used to examine the data for underlying ideas, patterns and assumptions about factors that might contribute to a large Q fever outbreak in Australia. Data familiarization was undertaken by reading and re‐reading the focus group transcripts, to identify commonalities found within the data. These commonalities were captured by generating codes consisting of a word or short phrase that assigned a summative attribute for a portion of language‐based data (Saldaña, 2015). Initial codes were considered for each state and combined to develop the coding framework. The coding framework produced by TT was crosschecked with another individual within the project team (LH) and discussed amongst the whole research team. Relevant data from the transcripts were then collated under each code in NVIVO 12. The coded data was evaluated for unifying features and organized into subthemes. Subthemes were clustered together under higher level themes that captured implicit topics covered by the data. Themes, subthemes and codes were reviewed in relation to each other and refined to ensure that they accurately described the meaning and identity of recurrent patterns and ideas presented by the participants. Quotes from the expert discussions are used to demonstrate the results of the analysis. Omissions or insertions are indicated by square brackets and used to increase intelligibility of the sentences. The original tenor has not been altered.

## 3 RESULTS

### 3.1 Sample characteristics

**FIGURE 1:**
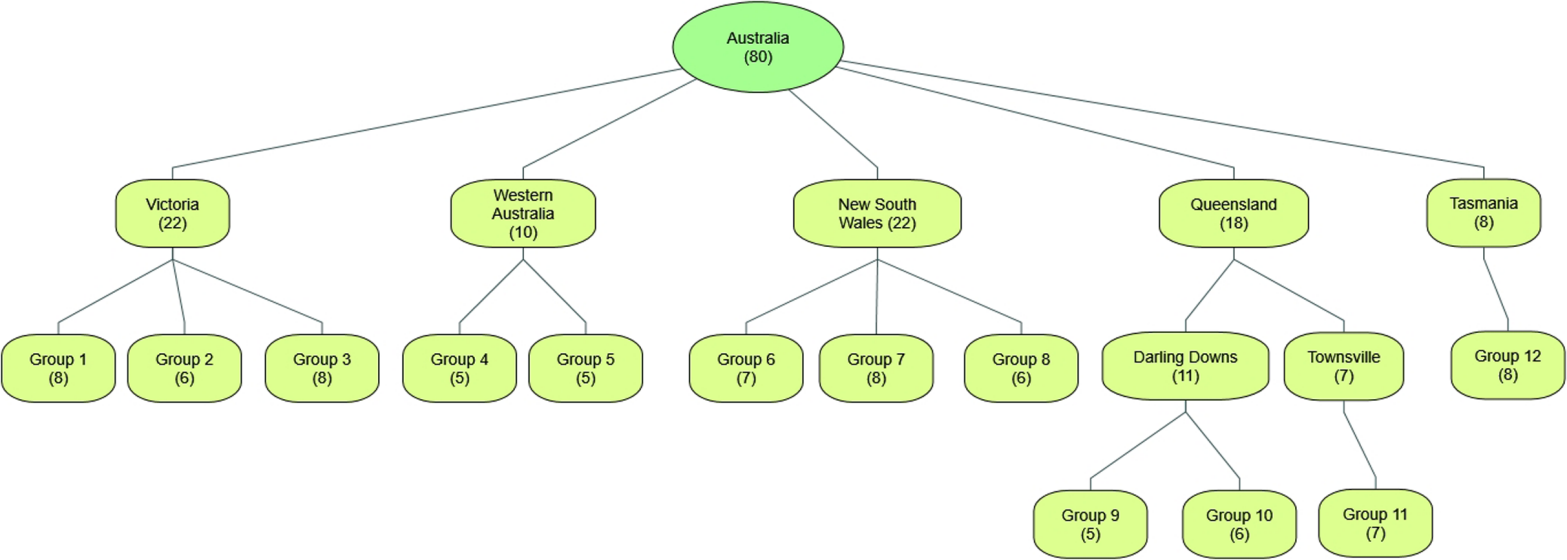
Distribution of participants by state and location with number of participants in each small group in brackets

Focus groups were held in five states (New South Wales, Victoria, Queensland, Western Australia and Tasmania) with 80 experts, 40 from human health/environmental health, 40 from animal health, who were divided into 12 small groups. Human health professionals were recruited from occupational safety regulators, public health units, environmental protection agencies, infectious disease physicians, laboratory scientists and academics. Animal health professionals were recruited from government veterinarians, meat and dairy industry representatives, farmer's federations, laboratory scientists and academics. Environmental health professionals have been grouped with those working in human health because it was difficult to distinguish their roles from those working in human health. The distribution of participants by profession and state are provided in Table 1 and Figure 1. The overall response rate of invitees was 48%.

**TABLE 1:** Distribution of participants by profession

Human health (40 participants)	Animal health (40 participants)
Worksafe (*n* = 4) Department of Health/Public Health Units (*n* = 23) Environmental health (*n* = 2) Infectious disease physicians (*n* = 4) Laboratory scientists (*n* = 3) Academics (*n* = 4)	Government veterinarians (*n* = 14) Private veterinarians (*n* = 3) Livestock Industry (*n* = 3) Abattoirs (*n* = 1) Farmer's Federation/Producers (*n* = 5) Academics (*n* = 14)

### 3.2 Thematic analysis

Participant discussions during the focus groups were centred on describing outbreak scenarios and factors that contribute to disease outbreaks. Analysis of this data provided insight into the evolving epidemiology of Q fever in Australia and resulted in the identification of putative risk factors for disease outbreaks. Large outbreaks affecting more than 25 people, were considered by the experts to be very uncommon events but could occur if ‘the right cluster of risk factors’ aligned to drastically amplify transmission and result in the ‘perfect storm.’ These observations demonstrate that an underlying mix of antecedent factors is necessary to create conditions conducive for the emergence of Q fever in a susceptible population. Whilst it is valuable to examine the effect of individual risk factors, they often do not occur in isolation. As part of this analysis, several scenarios are explored to understand how the convergence of any number of risk factors can result in an outbreak.

#### 3.2.1 Risk factors for Q fever outbreaks

The hierarchical relationship between risk factor themes, subthemes and codes are listed in Table 2. Risk factor themes, subthemes and codes have been created to capture patterns found in the data and serve as a framework to interpret and anticipate the threat of Q fever outbreaks in a variety of scenarios. However, they should not be considered mutually exclusive as there is a degree of overlap and interaction between the risk factors. For example, climate change and the presence of feral animals is influenced by human activities.

**TABLE 2:** Hierarchical relationship between risk factor themes, subthemes and codes. The number of groups that mention each code is also included

Theme	Subtheme	Code	Number of groups that mention the code
Anthropogenic	Disease awareness	Mitigation strategies (vaccination)	12
	Industry practices	Aggregation of animals Animal production system Transport, processing and distribution Workforce	12 12 12 12
	Land use	Urban encroachment Urban planning	9 7
	Human behaviour	Tourism and leisure Lifestyle and hobbies	10 12
Ecological	Physical environment	Geographical distribution Drought & climate change	12 12
	Agent dissemination	Environmental contamination Dispersal on dust, aerosol and wind	12 12
	Animal hosts	Species specific traits Parturition and birth products Feral animals	9 12 12

Two main themes that emerged from the data were anthropogenic and ecological risk factors. Anthropogenic risk factors encompass activities carried out by people that influence the risk of transmission of infection from animals to humans by promoting interaction between humans and animals (including animal products and waste) either directly (direct contact) or indirectly through a shared environment. Conversely, factors associated with the ecology of the disease relate to the biological characteristics of the agent and its interaction with the host and physical environment that affect its abundance, spread and distribution. Details about subthemes and codes for anthropogenic and ecological risk factors are described below.

##### Disease awareness

Lack of disease awareness was considered an important driver that increases the likelihood of infection because protective measures that prevent or reduce the incidence of infection are unlikely to be instituted. Participants emphasized vaccination as the ‘highest order’ of prevention that has been very effective at reducing the incidence of disease and outbreaks, particularly in abattoirs where workers are routinely immunized in compliance with occupational health and safety regulations. Unfortunately, disease awareness and consequently vaccination uptake were deemed inadequate in alternate settings, even amongst high‐risk groups working in animal industries.

The issue is [sheep are not considered] a traditional source, and I would suggest vaccination rates would be relatively low amongst this group and awareness would be low. I remember a bloke who was a stock agent. I said, "Have you got your Q Fever vaccine?" And he said, “No. I only handle sheep so that's not a problem.” So, there's not a perception that there is a risk amongst those sheep farmers. [Group 7]

Participants expect that members of the public are oblivious to Q fever and would therefore have no reason to seek vaccination even if they end up in high‐risk exposure situations. This lack of awareness makes them an especially vulnerable group.

Interesting that hobby farmers are often unaware of any of these issues, so they're sort of naive to the understanding of Q fever. And so, if you split up a big farm where you've got a farmer, understands the risk ‐ the farmer and family have all been vaccinated ‐ splits the farm up, lots of hobby farmers go in there, get a few cattle each, they are totally unaware of that risk because they're not farmers. They're businessmen from Sydney who are just dabbling. [Group 7]

Knowledge and understanding of Q fever risks can reduce susceptibility to infection when businesses and individuals seek immunization or other control measures such as hygiene and personal protective equipment.

##### Industry practices

Agricultural characteristics and industry practices that influence the occurrence of Q fever outbreaks were discussed at length by participants, which resulted in four risk factor codes being developed: (a) aggregation of animals, (b) animal production system, (c) animal transport, processing and distribution and (d) workforce.

##### Aggregation of animals

Aggregation describes the accumulation of large number of animals, sometimes from various sources, in a location, usually in high density and/or with a high throughput. Settings commonly described by participants included abattoirs, saleyards, shearing and feedlots/drought‐lots and relate predominantly to livestock. Animal shelters and veterinary hospitals that deal with dogs and cats were occasionally mentioned by participants.

Participants cited the presence of ‘a lot of people’ in these settings, ‘close proximity’ and ‘concentration of animals’ with ‘more opportunity for contact,’ combined with known cases and outbreaks associated with these environments as reasons for aggregation of animals being a driver of Q fever outbreaks.

Well, there's more opportunity to make contact [in a feedlot] because there’s more animals in close proximity to each other, and there’s more build‐up of waste in one location. [Group 6]

##### Animal production system

Descriptions of husbandry practices regarding the day‐to‐day care and rearing of animals are included under animal production system. Intensive livestock systems were repeatedly mentioned as a risk factor for Q fever transmission because high animal stocking rates exacerbates disease amplification and increased human contact promote disease transmission (Lindahl & Grace, 2015; Semenza et al., 2016).

And personally, I think the risk would increase as you get the more concentrated operations. You've got the risk of greater cow‐to‐cow transmission, feed‐ and environment‐to‐cow, and cow‐to‐environment transmission, and cow‐to‐person transmission. [Group 10]

Participants also noted that enterprises that practiced good biosecurity, hygiene and disease surveillance and used technology to automate activities such as robotic milking systems decreased the risk of disease transmission.

Type of animal production system also relates to the number of animals and farms at the national or state level and the number of animals within individual farming enterprises. Participants said that the larger the size of the animal population and number of workers involved in the system, the greater the risk of transmission.

You've got still a big risk. You've got big numbers of animals and of farms. And the number of people working in the industry right through from on‐farm, all your shearers, your service providers, transporters, abattoirs, saleyards. [Group 1]

##### Transport, processing and distribution of animals, animal products and waste

Participants considered that the transport, processing and distribution of animals, their products and waste increase the risk of transmission because it leads to the wider dissemination of pathogens and multiplies the number of people exposed to Q fever. Livestock were associated most with this risk factor due to frequent movement of these species onto processing facilities, such as abattoirs and saleyards, and the processing and distribution of livestock products, namely meat, milk and wool. Members of the public are exposed when major livestock routes pass through or close to populated areas and when commuters drive behind livestock trucks. The introduction of new animals onto a premise also increased the risk of disease spread. Some participants commented that the improper disposal of animal waste and effluent from farms and processing plants, and the use of manure as fertiliser by the community posed a risk.

(speaker a) So that's why your truckers and your saleyard workers and other people are going to be exposed that way. Musterers, abattoir workers. (speaker b) So, along the chain of processing, isn't it, from collection all the way to processing, yeah? [Group 9]

Animal products mentioned included dairy, wool, meat and hides. Unpasteurized milk was a recurrent concern in most groups because of pathogen survival in the untreated product and sale to the broader community.

I think unpasteurised goat's milk is also a major issue. In the past few years, I think there's been a lot of attention on illegal sale of unpasteurised goat's milk and I personally wouldn't be surprised if I heard that a Q fever outbreak [related to that]. [Group 7]

Raw kangaroo meat fed as pet food for dogs and cats was mentioned as a potential source of Q fever.

The work of the Sydney group found that the raw kangaroo meat that was being fed to animals, even domestic animals, was quite frequently contaminated with Coxiella. [Group 10]

##### Workforce

Workforce stability refers to the degree to which workers remained employed with an organization or in a location. It encapsulates comments about ‘stable’, ‘transient’, ‘casual’ and ‘itinerant’ labour within livestock industries.

‘Transient’ workers, like ‘backpackers’ or ‘foreign workers’ or places with a ‘high turnover of staff’ were identified at greater risk compared to ‘stable’ workplaces with long‐term staff. Participants identified that because ‘transient’ workers pass quickly through workplaces, such as abattoirs and farms, they were unlikely to be aware of the risks, vaccination or have had previous exposure. Furthermore, the issues and costs surrounding the vaccination process could deter businesses from getting their workers immunized particularly if many of them are only temporary.

The itinerant workforce that can happen on a sheep property, particularly in the wool industry, they can get a lot of backpackers in around shearing and crutching. And also the nature of sheep farmers. I'm not sure if SafeWork finds the same thing, but I find that sheep farmers are less likely to ‐‐‐ they're probably less likely to cop the money for vaccination. They run on a very thin margin a lot of the time, especially in the western areas. So, for me, when I look at sheep farmers within our association, I see them as a bigger risk than beef cattle farmers. [Group 7]

Contractors and ‘contract teams’ in the construction industry were also included under the category of ‘workforce’. Even though this group may be in stable employment, participants considered these workers to be at risk when they are deployed to operate in contaminated rural environments.

(speaker a) So not just the solar farms but also just new infrastructure where civil engineering‐‐ roads going through in areas so that's an area that we’ve been – done some targeted work in. (speaker b) Mining. Gas. All those industries where you're going onto rural land where there's not only cattle but kangaroos. (speaker a) So a whole cohort of workers that you wouldn't necessarily assume to be ‐‐‐ susceptible to Q fever, but they are because they're going into land that's historically used for [farming]— [Group 10]

Participants also noted the emergence of other occupations not normally considered to be high‐risk that could become a susceptible group if they involved novel work with animal products. Examples include fashion designers obtaining animal hides from a tannery or cosmetic factories processing sheep placentas.

##### Land use

Patterns of land use that place naïve human populations in proximity with livestock industries and wildlife habitats increases the overlap between animals and people in the environment, and the potential exposure to Q fever. Urban encroachment and urban planning were specific codes identified in the data that relate to the concept of land use. They represent the intersection between natural and built environments, public interests, industrial activities and policies.

##### Urban encroachment

Participants discussed that urban encroachment is driven by population growth and the desire for alternative lifestyles. This resulted in the transformation of agricultural land and native habitats into urban centres and residential spaces that forces people, wildlife and livestock closer together. Recurrent examples are the development of lifestyle blocks on urban fringes and the subsequent exploitation of green feed on household lawns and public spaces by macropods living in nearby forests or bush.

I think a big thing that we consider is urban sprawl because if we're pushing society on to the fringes and then they're coming in contact with wildlife. Wildlife will play a bigger role, but also when we're moving farms to where wildlife previously had their habitat, wildlife and domestic animals are going to be coming in to contact more. So inevitably wildlife [are] going to play a much bigger role. And that's I think what we've seen with a lot of re‐emerging zoonosis. [Group 4]

##### Urban planning

Urban planning involves the design and development of human settlements and communities. This includes urban and suburban zones as well as agricultural land and conservation parks in rural and regional areas. This risk factor arose through several comments about saleyards, abattoirs or feedlots that are high‐risk activities for Q fever, being constructed right up close to naïve populations in residential areas.

We also get a lot of complaints ‐ with poor development planning decisions in our region. We've had the two in the last two weeks, actually, two different feedlots. So, people were there in the house, the next minute, a feedlot turns up and digging all the dust and—we always talk about Q fever, and maybe they need to think about getting vaccination [because of] that poor development. [Group 10]

Participants felt that there was opportunity for improvement in urban planning policies to widen the buffer zone and prevent facilities such as abattoirs or saleyards being built too close to housing.

##### Human behaviour

Human behaviour is expressed as individual or group action and interaction that is influenced by culture, personal values and attitudes. Infectious disease can result from behaviour that increases exposure to the pathogen. The two specific risk factors that emerged under human behaviour are further described below.

##### Lifestyle and hobbies

Participants described numerous situations in which members of the public have close and recurrent contact with livestock and wildlife because of lifestyle choices or hobbies. This includes comments about hobby farms, small holdings or backyard farmers and wildlife carers where people live in close association with these animals. Additional activities like ‘backyard slaughter’ and ‘hunting’ of wildlife and feral animals was also identified to potentially increase the risk of transmission.

The worst‐case scenario would be a smallholder being very generous to his neighbours and friends and ordering a couple of goats and distributing the meat to people ‐‐ not out of the question at all. [Group 12]

##### Tourism and leisure

Tourism is the activity of people travelling to and staying in places outside their usual environment for leisure and recreation. This risk factor was identified from discussions about public attendance at zoos, animal parks, sanctuaries, animal shows, races and camp drafts and camping in nature. Groups noted the increasing popularity of tourist visits to saleyards and short‐term stays at farms that have opened to the public. Other unusual activities also discussed were interactions with kid goats whilst practicing yoga and music festivals that are held on farmlands. Participants were concerned that these situations pose a significant Q fever threat because tourism enables immunologically naïve members of the community access to potentially risky environments where wildlife and livestock are aggregated.

[…] people going to caravan parks over Christmas, where the kangaroos hop all around their campsites. It makes you wonder why we're not seeing big outbreaks amongst those groups of people. […] And in our main caravan park, everyone's got their tents and they [flap] their tents and everything around and [their things] everywhere. That's where I'm waiting to see one of those outbreaks […]. [Group 6]

More dairy farms allow or encourage members of the public to come on‐site to look at the dairy cows so there's more people ‐‐ members of the community having more contact with dairy environments. [Group 10]

##### Physical environment

Environmental factors refer to extrinsic physical factors such as geography and climate that affect the agent and increase the opportunity for exposure, which predisposes to disease.

##### Geographical distribution

Location was identified as a factor influencing disease incidence, with participants stating that Q fever incidence is unevenly distributed across Australia. Queensland and New South Wales have a higher incidence of disease compared with other states, with well recognized ‘hotspots’ for infection. Geographical variation in disease occurrence also relates to the difference in numbers and distribution of the animal species across the states. If an animal species is not present in a location, then it is unlikely to be the source of an outbreak.

I was just thinking about where we see Q fever. It's not in this distribution where these big wool sheep farms are living. In Tasmania, Victoria and south [Western Australia] is not the Q fever hotspots. It makes me think that maybe the beef, the cows are in those area where we know there's the environmental burden and ‐‐‐ the New South Wales, Queensland Coast they are more likely [to have Q fever]. [Group 1]

##### Drought and climate change

Climate can directly impact disease transmission through its effects on the ecology, survival and spread of the pathogen. Participants said that drought exacerbated the risk of Q fever because it promotes dry and dusty conditions and increases the overlap between livestock, wildlife and humans. Dry conditions lead to feed and water shortages, drawing macropods (kangaroos and wallabies) closer to human settlements and increasing the transportation of livestock through urban and regional areas for slaughter or agistment. Participants anticipated that incidence of drought conditions will increase with climate change.

Also, the seasonal conditions. It's the same with sheep. If we're in a drought, there's more movement of cattle and sheep to processing plants and to saleyards, so there's a bigger risk of them spreading bacteria through country towns. [Group 7]

##### Agent dissemination

Environmental contamination, wind‐borne spread, dust and aerosols are risk factors that magnify agent dissemination and are interconnected with physical environment factors.

##### Environmental contamination

Environmental contamination was considered an important source of infection, even in the absence of livestock or wildlife hosts, because of the organism's ability to accumulate and remain infective in the environment for a long time.

But the people who contracted [Q fever] were the grounds people and the people at risk were the grounds people and their families. Bearing in mind that they get covered in dust when they're doing this industrial lawn mowing so it all comes back to issues mentioned before; contamination of the soil from who knows what, but presumably some macropod groups do that. [Group 7]

##### Dispersal on dust, aerosol and wind

Dry, dusty conditions or aerosolization of contaminated particles were frequently mentioned together with wind‐borne spread that enables dispersal of the organism over an extensive area and increases the risk of widespread exposure.

The aerosol thing is quite important. Considering some of the wind we get in Western Australia, in summer, you certainly‐‐ the ground gets very dry. You end up with‐‐ and I've been on farm. And if somebody's doing something with livestock on the next farm, and then you see this cloud of dust moving across the horizon. [Group 5]

##### Animal hosts

This subtheme encompasses specific features that participants described as peculiar to the animal host and include species‐specific traits, parturition and feral animals.

##### Species‐specific traits

Species‐specific traits are intrinsic to the animal host and determine susceptibility and response to the causative agent. Species‐specific traits include participant accounts of higher infection rates amongst some species, seroprevalence or shedding studies, and accounts of the species being implicated in known human outbreaks. For example, dairy goats as a species are considered highly effective amplifiers and spreaders of the Q fever pathogen.

Most of the outbreaks that we've seen in the world have been associated with dairy goats, so. And also, dairy goats tend to have a higher risk of abortion compared to other animals if they're infected. So, that leads to more environmental contamination and the risk of associated humans getting infected. So, I think yes, dairy goats have to be up there close to the top I think. [Group 8]

##### Parturition and birth products

It was well known amongst participants that infected animals shed high numbers of the bacteria in products of parturition. Increased risk was therefore associated with ‘parturition’, ‘abortion’, ‘birthing’, ‘birth products’, ‘placentas’, ‘foetal fluids’ and ‘neonates’.

Yes, goats are way bigger shedders particularly when they give birth. So, they're the ones who do absolutely abort as a result of it. So, when they do abort they shed enormous amounts of the bacteria. So, you know it's just much higher than what faecal spread would be. [Group 11]

Animal species with greater human involvement during parturition were considered the highest risk; dairy cattle with seasonal calving patterns and situations where herd managers and/or their veterinarians provided assistance to individual cows at the time of calving. Cat breeders received specific mention as being at risk of Q fever.

##### Feral animals

Feral animals are non‐native species that have escaped or been released from domestic or captive status and are living more or less as a wild animal. Participants suggested that feral animals (cats, deer, goats and pigs) could be reservoirs of Q fever but there was uncertainty about their relative importance as a reservoir because of a lack of peer‐reviewed empirical research. Feral goats and pigs were discussed the most frequently because pig hunting is common and feral goats are regularly harvested in large numbers for meat. The harvesting of feral goats has been connected with known outbreaks.

So, there's definitely been outbreaks with people infected and even dying, with feral goat association. Yeah, so that's a good point, not knowing the prevalence in the goats themselves. [Group 2]

#### 3.2.2 Scenarios

Descriptions of scenarios were used by participants to generate and explore ideas about the causal relationship between risk factors and outbreaks. The scenarios presented in Table 3 are adapted from the discussions to provide tangible examples of real and plausible outbreaks. Scenarios show that it is possible for the same mix of risk factors to lead to an outbreak within multiple different settings. In this way, scenarios illustrate the multiple dimensions through which a complex accumulation of risk factors can lead to disease emergence.

**TABLE 3:** Examples of real and proposed Q fever outbreak scenarios with associated risk factors

Scenario	Risk factor (subtheme)	Risk factor (codes)
The very large outbreak in The Netherlands associated with dairy sheep and goats that affected thousands of people in surrounding districts (real)	Disease awareness Industry practices Land use Agent dissemination Animal hosts	Animal production system; species specific traits; urban planning; dispersal on dust; aerosol and wind; mitigation strategies
Many cases detected among attendants at music festivals and sporting events organised near or on farmland where reproductive issues have been previously noted in small ruminant herds/flocks (proposed)	Disease awareness Land use Human behaviour Agent dissemination Animal hosts	Animal production system; species specific traits; lifestyle and hobbies; environmental contamination; mitigation strategies
Outbreak among visitors to a dairy sheep farm during the busy school holidays There is a restaurant and farm shop on‐site The farm promotes public viewing of their operations and ewes during lambing (proposed)	Disease awareness Human behaviour Animal hosts	Tourism and leisure; parturition and birth products; mitigation strategies
Outbreak among recently employed foreign workers in a goat abattoir (real)	Disease awareness Industry practices Animal hosts	Aggregation of animals; transport, processing and distribution; workforce; species specific traits; mitigation strategies
Outbreak amongst staff volunteering in an animal shelter after a feral cat gave birth (real)	Disease awareness Industry practices Animal hosts	Aggregation of animals; workforce; parturition and birth products; feral animals; mitigation strategies
Outbreak among staff and visitors at a research and training facility following a workshop using pregnant sheep as models for teaching in surgical techniques (proposed)	Disease awareness Animal hosts	Parturition and birth products; mitigation strategies
Outbreak among students and staff at a university following the mowing of a campus lawn frequented by kangaroos during the drought period (proposed)	Disease awareness Land use Physical environment Agent dissemination	Urban encroachment; drought and climate change; environmental contamination; mitigation strategies

Participants often described situations in which members of the public, not generally considered to be at high risk, were exposed to infection and proposed that Q fever acquired in the community, outside of traditional‐high‐risk groups in occupational settings, is becoming an increasingly recognized public health problem. Comments regarding acquisition in the community (114 codes) were more numerous than occupational‐acquisition (92 codes).

Participants articulated that outbreaks of Q fever are very complex and have ‘required substantial efforts to understand and control’. Limited knowledge of disease epidemiology and the interaction of diverse known and unknown factors make it difficult to correctly predict the likely source of outbreaks:

So, I think it's unlikely, but Q fever continues to shock me and surprise me. So [you're never] going to say impossible. [But] we don't see it from ingestion yet [laughter]. So that's why I sat there and said nothing because I don't feel like ‐‐‐ the more I know, the more I read and know about Coxiella, the more I realise I know nothing [laughter]. [Group 6]

## 4 DISCUSSION

The aim of this study was to use expert opinion to better understand how Q fever outbreaks are perceived to occur in Australia and provide commentary on to how various factors might drive Q fever transmission and contribute to future outbreaks. The data collected was highly dependent on the experts that participated in this study and may not reflect the opinion of all Q fever experts in Australia. However, the purpose of this study was to record the conversations around Q fever outbreaks rather than individual viewpoints. Expert opinion focus groups enabled interaction between participants for the exchange of information and experiences as well as the linking of concepts and generation of novel knowledge that may not be achieved with individual interviews alone (Braun & Clarke, 2013; Nagle & Williams, 2013; Tong et al., 2007). Multiple expert perspectives broaden awareness of the varied ways in which incident Q fever cases occur and has enabled the documentation of contributing factors that are not necessarily described in the published literature. These latent variables cannot be directly observed or measured but underly the existence of other observable risk factors and are inferred from the description of various outbreak scenarios. Analysis of the scenarios presented in the focus groups provided a multidimensional understanding of risk factor dynamics, were useful for anticipating future disease events, tracking the evolution of outbreaks and the changes that magnify their occurrence. This process of hazard identification and evaluation of Q fever outbreaks provides a solid foundation on which to build risk mitigation measures.

A significant outcome of this study was the identification of perceived risk factors that contribute to Q fever outbreaks in Australia. Large Q fever outbreaks can occur when an underlying mix of antecedent risk factors combine to create ‘perfect storm’ conditions for a pathogen to emerge in susceptible populations. Sixteen risk factor codes, seven risk factor subthemes (disease awareness, industry practices, land use, human behaviour, physical environment, agent dissemination and animal hosts) and two main risk factor themes (anthropogenic and ecological) were generated from inductive thematic analysis of repeated patterns in the data. There was consistency across the groups regarding the variables that were considered to contribute to Q fever outbreaks. This consensus amongst participants suggests that a common knowledge exists for aspects of the disease epidemiology and provides confidence that despite limitations to our understanding of the epidemiology of Q fever in Australia, the identified drivers and risk factors merit assessment and, where possible, management for the prevention of Q fever outbreaks.

It is well recognized that many zoonotic disease outbreaks around the world have occurred because of factors related to human activity and behaviour. Anthropogenic drivers similar to the ones highlighted in this paper (e.g. trade, population expansion, urbanization, tourism) have been described extensively in other countries (Lederberg et al., 2003; Lindahl & Grace, 2015; Olson et al., 2015; Prowse, 2010; Semenza et al., 2016). Fundamentally, human action directly or indirectly increases the likelihood of transmission through intensive agricultural practices that amplify infection and shedding by animal hosts, by moving infected animals to previously uninfected, uncontaminated areas or enabling access for susceptible humans to places where they can encounter infected animals or contaminated environments (Lindahl & Grace, 2015; Prowse, 2010; Sabin et al., 2020; Semenza et al., 2016).

Risk factors classed as anthropogenic were the most numerous in this study and are the most obvious targets to evaluate for intervention. There is better comprehension of the roles that human activities play in outbreaks and there is greater potential to manipulate human factors compared to the ecological factors that are intrinsic features of disease epidemiology. For example, environmental conditions that promote wind‐borne dispersal of the organism can only be monitored but not prevented (Tissot‐Dupont et al., 2005). Anthropogenic risk factors for Q fever outbreaks identified in this study include disease awareness, industry practices, land use and human behaviour. It is important to recognise that many anthropogenic risk factors are correlated with desirable activities required by society such as recreation, lifestyle, agriculture, business and trade (Lindahl & Grace, 2015). Therefore, when evaluating these risk factors for intervention to reduce Q fever exposure, balancing the benefits and risks of such activities could be a major issue when they are in conflict with public health interests.

Risk factors associated with the livestock industry were the most numerous and were intensively discussed by all focus groups. This is not surprising considering that livestock production is a major industry in Australia worth more than USD$ 21 billion (2021) annually and comprises many millions of head of stock (Australian Bureau of Statistics, 2016). The larger number of industry risk factors may also be attributable to the way in which expert opinions were elicited in the group exercises using industry specific species to estimate risk. Increasing numbers of animals reared in high‐intensity, high‐density production systems is often an important factor in zoonotic disease emergence (Lederberg et al., 2003; Prowse, 2010).

Clearly, industry factors not only pose a risk to those working in the livestock sector but also to the wider community and even in urban populations that are usually considered low risk. However, these identified risk factors are essential functions of livestock industries and any control measures instituted need to account for both industry and public health interests. Difficulties in balancing these competing interests is exemplified in the control measures applied during the 2007–2010 outbreak of Q fever in The Netherlands. Authorities instituted drastic measures of breeding bans and the pre‐emptive culling of thousands of pregnant does in a frantic effort to limit transmission to humans, which may have unnecessarily inflated the number of livestock destroyed (Roest et al., 2011; Wayop et al., 2011). Although this approach was successful at curtailing the epidemic, the necessity of depopulation efforts that ruined the Dutch dairy goat industry remains contentious (Plummer et al., 2018). Pre‐empting zoonotic disease outbreaks can facilitate the development of strategies ahead of the event to reduce avoidable loss of life and harm to the livestock industry whilst protecting public health (WHO, 2015). At the very least, livestock industries can be prepared well in advance for the possibility that heavy‐handed control measures may be applied during times of tension.

It is evident from analysis of the group discussions that the role of farms, livestock industries and agricultural spaces are shifting and expanding into areas beyond their original purpose. Descriptions by participants of tourist visits to farms and saleyards, sporting or music events held on farms, petting zoos, farm stays, farm shops and cafes all relate to concepts of agritourism. Agritourism is defined as a form of agricultural diversification in rural communities where working farms develop practices to attract visitors for the purposes of educating the public, enjoyment of visitors and to supplement farm income (Amsden & McEntee, 2011; Ecker et al., 2010; Phillip et al., 2010; Wayop et al., 2011). Similarly, changing land use relates to descriptions of urban planning policies that permit abattoirs or saleyards to be built close to existing residential areas and urban encroachment onto farmland and native habitats. Both agritourism and changing patterns of land use have been shown to increase the risk of Q fever exposure and outbreaks. Certainly, open farm ‘lamb‐viewing days’, farmers markets and petting zoos have been implicated in previous Q fever outbreaks amongst visitors (Porten et al., 2006; Valkenburgh et al., 2011; Whelan et al., 2012). In South Australia, the greatest number of laboratory confirmed notifications recorded between 2007 and 2017 were in a suburb containing an abattoir (Rahaman et al., 2020). Those living on large lifestyle blocks bordering farmlands and native forests in Northern Queensland have been identified as having an increased risk of Q fever, attributed to wildlife encroaching on domestic properties and excreting *C*. *burnetii* in the environment (Sivabalan et al., 2017).

Changing land use, diversification of livestock enterprises, emerging industries and alternative lifestyle trends function to combine spaces that were principally designed for work and primary production with social activities such as farm‐based agritourism, home‐based hobby farming and community‐supported agriculture (Amsden & McEntee, 2011). This conflation of agriculture with recreation and leisure points to emerging social and lifestyle factors that are widening the categories of people exposed to Q fever. These trends have largely been driven by the increasing desire of urbanized populations to connect to farms as well as recent coordinated regional approaches to develop agritourism as a strategy for growth of agricultural businesses and rural communities (Ecker et al., 2010). Those seeking alternative lifestyles on the rural‐urban fringe often keep small numbers of livestock on ‘hobby farms’ purely for recreational pursuit and without expectation of being a primary source of income (Amsden & McEntee, 2011). More often than not, people engaging in agricultural activities predominantly for socio‐cultural reasons have little or no understanding of the physical and biological risks (Prowse, 2010; Wayop et al., 2011). Q fever is likely an under‐recognized hazard and it could be argued that the people making decisions around risk within these settings have prejudices that are skewed towards complacency, or at least away from knowledge of disease. Regulations to protect the public against zoonotic diseases, inclusive of Q fever, may not be keeping up with trends in land use and human behaviour, resulting in the potential for increased risk of Q fever infection in the community outside of traditional at‐risk occupations.

One risk factor common to all real and potential outbreak scenarios is the absence of ‘disease awareness’ amongst susceptible people whereas other risk factors recur variably. Because it is a constant for all situations, improving disease awareness to reduce susceptibility amongst the population could be the most successful strategy for Q fever control. Moreover, the other anthropogenic risk factors (industry practices, land use and human behaviour) are inherent to social aspirations or are necessary functions of livestock industries, which make them impractical or difficult to amend. Awareness is the critical first step for the control of disease transmission and should be promoted so that people engaging in high‐risk activities or environments can take precautions such as basic hygiene measures and/or vaccination (Eastwood et al., 2018). In a companion study (unpublished), when experts were asked to list and rank important preventative measures for Q fever, all groups were unanimous that vaccination of people was the most effective approach, followed by education and awareness, and then policy around urban and regional planning.

Q fever is a vaccine preventable disease but the vaccine (Q‐VAX^®^) is only licenced for use in Australia. Australian public health authorities have been successful in using vaccination programmes to reduce the number of cases and outbreaks in high‐risk environments such as abattoirs (Brotherton et al., 2007; Communicable Diseases Network Australia, 2018). Immunization is recommended for all people who are likely to be exposed to *C*. *burnetii* (Brotherton et al., 2007) but it is not always easy to discern infection risks and impose vaccination protocols outside of conventional risk settings and in non‐occupational environments. Not only is the incentive to get immunized low because awareness and risks of infection are poorly understood by the individual and medical practitioners, but high costs, limited availability and rigorous pre‐vaccination assessment can present further barriers for vaccination uptake (Eastwood et al., 2018; Rahaman et al., 2019).

Other approaches to manage the biological risk are through veterinary, environmental and sanitary interventions that reduce the bacterial load in the environment and minimize human exposure to *C*. *burnetii*. This strategy was employed in the 2007–2010 Netherlands outbreak where vaccination of livestock, proper disposal of biological materials and disinfection were made compulsory for all dairy sheep and goats including small ruminants on farms offering recreational activities (Hogerwerf et al., 2011; Vellema & van den Brom, 2014; Wayop et al., 2011). Vaccination of livestock is a valuable tool that provides benefits to humans by controlling infection and shedding in the animal source (Rahaman et al., 2019). In the absence of a commercial vaccine for livestock there is increased the reliance on other management measures as hygiene and biosecurity to control infection. A further complication to the control of environmental contamination and transmission is the presence of *C*. *burnetii* in native and feral wildlife species that may act as additional reservoirs of infection (Eastwood et al., 2018; Garner et al., 1997; Pope et al., 1960). In lieu of vaccinating animals, public farms and animal sanctuaries should provide visitor warnings and explanations about zoonotic diseases, supply good hand washing facilities and instructions for general hygiene measures, keep animals away from human drinking and eating areas and keep parturient animals away from visitors (Wayop et al., 2011).

Unfortunately, there are many historical examples where public health policies for the control of Q fever have been developed only after the occurrence of serious outbreaks in humans (Md Rezanur Rahaman et al., 2019; Wayop et al., 2011). Consulting with multidisciplinary professionals to anticipate outbreaks could greatly improve outbreak management by shifting the approach from being reactive, in which measures are developed and enacted only when the outbreak is already underway, to one of anticipation, where health system tools and strategies are already in place to counteract the disease event (Dallas et al., 2019; WHO, 2015). The emerging scenarios and numbers of human cases acquired outside of traditional risk settings is expanding the view of Q fever from being exclusively an industry related disease to a potentially wider public health issue (Australian Technical Advisory Group on Immunisation (ATAGI), 2018). This expansion is driven by greater overarching economic, political and socio‐cultural forces that influence human behaviour and help explain the occurrence of anthropogenic risk factors.

Disease outbreaks are complex phenomena that involve the convergence of diverse elements, not just that of the pathogen and host, but also the physical, political and socioeconomic environments in which they interact. Because Q fever is a disease with human, animal and environmental interfaces, a multidisciplinary One Health approach that involves multiple key stakeholders is recommended to provide a strong framework to handle the challenges of mitigating outbreaks (Rahaman et al., 2019; WHO, 2015). As the threat of Q fever outbreaks is evolving and largely driven by alterations in human behaviour, it could be considered a social problem as much as a medical one (WHO, 2015). Public health strategies based on traditional interventions such as biosecurity, sanitary measures and targeted vaccination of at‐risk occupational groups through workplaces are limited and difficult to implement when addressing the emerging risk in a wider community. Increasing focus should be placed beyond the conventional health system and on engaging with communities and individuals so that they become an integrated part of outbreak prevention and preparedness. The recent COVID pandemic has provided an opportunity to capitalize on enhanced awareness and knowledge in the community around infectious diseases. Preventive community health training that strengthens education and communication should be viewed as a long‐term programme, not only during an outbreak, for disease control measures to be effective (WHO, 2015). Further consultation with experts is required to define the responsibilities of various players, barriers to responsive actions and to understand the role of the community in combating outbreaks (WHO, 2015).

Q fever is an under‐recognized, complex disease in which the increasing convergence of risk factors will continue to amplify transmission and drive disease emergence. It is important to anticipate outbreaks by identifying hotspots of infection, interpreting known risk factors, analysing biological, ecological and behavioural drivers to concentrate appropriate resources and efforts into specific risk settings (WHO, 2015). The results from this study that identify perceived anthropogenic and ecological risk factors were expected and have been documented by various works addressing Q fever and other zoonotic diseases. However, the value of this work is in collating the expert knowledge across species and systems into a single assessment, which shows how risk factors converge to amplify transmission and drive the emergence of Q fever outbreaks. Descriptions of various scenarios in which the risk factors culminate indicate a growing awareness and concern around the risk of acquisition and outbreaks in the community, outside of traditional at‐risk occupations, due to emerging social values, trends and pressures that conflate agriculture with lifestyle and recreation. Knowledge of these risks and complex interactions should be used to support a move from a fragmented health‐centred approach to an integrated multidisciplinary approach for combating Q fever that engages with an increasing number of stakeholders and strengthens multisectoral preparedness (WHO, 2015). When reviewing conventional approaches for outbreak preparedness, at‐risk populations and the communities to which they belong should be viewed as part of surveillance and response. There are consequences associated with omission of these stakeholders, which highlights the need to utilize these key groups in the development of interventions. Effective mitigation strategies should be constructed considering the broader context and complexity of the scientific, economic, political and socio‐cultural aspects that intersect to drive outbreaks of this disease.

## CONFLICT OF INTEREST

The authors declare no conflict of interest regarding this study.

## Supporting information

Supplementary Material

## Data Availability

Research data are not shared.
