# Supplementary material for: Identifying scenarios and risk factors for Q fever outbreaks using qualitative analysis of expert opinion
Source: Zoonoses Public Health. 2022 Mar 3;69(4):344–58. doi: 10.1111/zph.12923 (PMC9310758; doi:10.1111/zph.12923)
Supplement: Supplementary file 1 — Supplementary Material [file ZPH-69-344-s001.docx]

# Expert opinion focus group worksheet template

**SPECIES-SPECIFIC RISK ESTIMATES**

**Scenario:** An outbreak of Q fever has occurred in people. At this stage there are no geographical descriptors or other epidemiologically defining features of the outbreak, although it is known to have affected more than 25 people.

Given that an outbreak has occurred, please **estimate the probability that the source of the outbreak was each of the following species** (each of which we know to have the capacity to carry *Coxiella burnetii*).

n.b. for each estimate, please complete the following:

a) Identify, using three crosses placed on the line, the **minimum**, **most likely** and **maximum** estimate, where 0 is impossible and 1 is a certainty.

b) Identify the confidence in your estimate (on a scale of 1 – 10)

c) Identify up to five factors that influenced this estimate (rank in order from most to least influential)

**Dairy cattle**

|  |
| --- |

1

(Certainty)

(Impossible)

0

|  |  |  |
| --- | --- | --- |

Confidence:

| 1. |
| --- |
| 2. |
| 3. |
| 4. |
| 5. |
| Other: |
|  |

Factors influencing your estimate:

**Beef cattle**

|  |
| --- |

1

(Certainty)

(Impossible)

0

|  |  |  |
| --- | --- | --- |

Confidence:

| 1. |
| --- |
| 2. |
| 3. |
| 4. |
| 5. |
| Other: |
|  |

Factors influencing your estimate:

**Dairy Sheep**

|  |
| --- |

1

(Certainty)

(Impossible)

0

|  |  |  |
| --- | --- | --- |

Confidence:

| 1. |
| --- |
| 2. |
| 3. |
| 4. |
| 5. |
| Other: |
|  |

Factors influencing your estimate:

**Meat and Wool Sheep**

|  |
| --- |

1

(Certainty)

(Impossible)

0

|  |  |  |
| --- | --- | --- |

Confidence:

| 1. |
| --- |
| 2. |
| 3. |
| 4. |
| 5. |
| Other: |
|  |

Factors influencing your estimate:

**Dairy Goat**

|  |
| --- |

1

(Certainty)

(Impossible)

0

|  |  |  |
| --- | --- | --- |

Confidence:

| 1. |
| --- |
| 2. |
| 3. |
| 4. |
| 5. |
| Other: |
|  |

Factors influencing your estimate:

**Meat Goat**

|  |
| --- |

1

(Certainty)

(Impossible)

0

|  |  |  |
| --- | --- | --- |

Confidence:

| 1. |
| --- |
| 2. |
| 3. |
| 4. |
| 5. |
| Other: |
|  |

Factors influencing your estimate:

**Pig**

|  |
| --- |

1

(Certainty)

(Impossible)

0

|  |  |  |
| --- | --- | --- |

Confidence:

| 1. |
| --- |
| 2. |
| 3. |
| 4. |
| 5. |
| Other: |
|  |

Factors influencing your estimate:

**Horse**

|  |
| --- |

1

(Certainty)

(Impossible)

0

|  |  |  |
| --- | --- | --- |

Confidence:

| 1. |
| --- |
| 2. |
| 3. |
| 4. |
| 5. |
| Other: |
|  |

Factors influencing your estimate:

**Dog**

|  |
| --- |

1

(Certainty)

(Impossible)

0

|  |  |  |
| --- | --- | --- |

Confidence:

| 1. |
| --- |
| 2. |
| 3. |
| 4. |
| 5. |
| Other: |
|  |

Factors influencing your estimate:

**Cat**

|  |
| --- |

1

(Certainty)

(Impossible)

0

|  |  |  |
| --- | --- | --- |

Confidence:

| 1. |
| --- |
| 2. |
| 3. |
| 4. |
| 5. |
| Other: |
|  |

Factors influencing your estimate:

**Wildlife**

|  |
| --- |

1

(Certainty)

(Impossible)

0

|  |  |  |
| --- | --- | --- |

Confidence:

| 1. |
| --- |
| 2. |
| 3. |
| 4. |
| 5. |
| Other: |
|  |

Factors influencing your estimate:

**Wildlife – harvested**

|  |
| --- |

1

(Certainty)

(Impossible)

0

|  |  |  |
| --- | --- | --- |

Confidence:

| 1. |
| --- |
| 2. |
| 3. |
| 4. |
| 5. |
| Other: |
|  |

**Other __________________________________**

|  |
| --- |

1

(Certainty)

(Impossible)

0

|  |  |  |
| --- | --- | --- |

Confidence:

| 1. |
| --- |
| 2. |
| 3. |
| 4. |
| 5. |
| Other: |
|  |

Factors influencing your estimate:

**Other __________________________________**

|  |
| --- |

1

(Certainty)

(Impossible)

0

|  |  |  |
| --- | --- | --- |

Confidence:

| 1. |
| --- |
| 2. |
| 3. |
| 4. |
| 5. |
| Other: |
|  |

Factors influencing your estimate:

**Other __________________________________**

|  |
| --- |

1

(Certainty)

(Impossible)

0

|  |  |  |
| --- | --- | --- |

Confidence:

| 1. |
| --- |
| 2. |
| 3. |
| 4. |
| 5. |
| Other: |
|  |
